# Antiplatelet strategy for patients with acute coronary syndrome and chronic kidney disease: a systematic review and meta-analysis

**DOI:** 10.3389/fcvm.2025.1527667

**Published:** 2025-02-20

**Authors:** Siqi Li, Dayang Wang, Xiaowan Han, Diying Zhang, Hongxiao Deng, Guozhong Pan

**Affiliations:** ^1^ Dongzhimen Hospital, Beijing University of Chinese Medicine, Beijing, China; ^2^ Second Department of Cardiology, Dongzhimen Hospital, Beijing University of Chinese Medicine, Beijing, China; ^3^ Institute of Cardiovascular Diseases, Dongzhimen Hospital, Beijing University of Chinese Medicine, Beijing, China

**Keywords:** dual antiplatelet therapy, acute coronary syndrome, chronic kidney disease, ticagrelor, clopidogrel

## Abstract

**Background:**

Patients with both acute coronary syndrome (ACS) and chronic kidney disease (CKD) face heightened risks of adverse cardiovascular events and bleeding. An optimal antiplatelet strategy for this patient population is needed.

**Methods:**

We conducted a systematic review and meta-analysis to evaluate comparative advantages of clopidogrel vs. ticagrelor in the choice of dual antiplatelet therapy (DAPT) strategies for patients with ACS combined with CKD, while also exploring the appropriate duration of DAPT in the presence of CKD. Relevant studies were retrieved from PubMed, Embase, Cochrane Library, and Web of Science. The primary outcomes were all-cause mortality, major adverse cardiovascular events (MACE), and major bleeding. Data were analyzed using RevMan 5.4.1, and STATA 14 was used to assess publication bias. This study was registered with PROSPERO (CRD42024593764).

**Results:**

Six studies involving 9,947 patients met the inclusion criteria. Compared with clopidogrel, ticagrelor was associated with a reduced risk of MACE (RR: 0.89, 95% CI: 0.80–0.99; *P* = 0.04) and stroke (RR: 0.66, 95% CI: 0.45–0.96; *P* = 0.03) in patients receiving DAPT. No significant differences were observed in all-cause mortality, major bleeding, cardiovascular death, or acute myocardial infarction. Three studies on DAPT duration showed a consistent trend, indicating that shortening DAPT duration did not benefit patients.

**Conclusions:**

In patients with ACS combined with CKD, ticagrelor-based DAPT has advantages over clopidogrel-based DAPT, which is associated with a lower incidence of MACE. And shortening the duration of DAPT does not improve clinical outcomes.

## 1 Introduction

In patients with acute coronary syndrome (ACS), the use of aspirin and P2Y12 receptor antagonists for dual antiplatelet therapy (DAPT) was recommended by clinical guidelines (1). Compared to single antiplatelet therapy, DAPT provides a stronger inhibition of platelet aggregation, thereby reducing ischemic events associated with ACS (1). However, long-term DAPT can significantly increase the risk of bleeding (2). The standard duration of DAPT for ACS patients remains 12 months. Patients who remain event-free and do not have a high bleeding risk after 3–6 months may be transitioned to single antiplatelet therapy, while those at high bleeding risk may switch to single antiplatelet therapy after 1 month of DAPT (1). However, the benefits of different anticoagulation strategies in ACS patients with multiple comorbidities are still unclear, particularly in cases accompanied by chronic kidney disease (CKD) (3).

CKD is an independent risk factor for the incidence and mortality of cardiovascular disease. When estimated glomerular filtration rate (eGFR) is <60 ml/min/1.73 m^2^, the risk of cardiovascular disease begins to increase, and it rises sharply when eGFR is <45 ml/min/1.73 m^2^, ultimately becoming the most common cause of death in patients with end-stage renal disease (4). In fact, regardless of the antiplatelet therapy regimen, patients with CKD have an approximately 3.5-fold increased risk of bleeding compared to patients without CKD (5). At the same time, these patients also have a high risk of thrombosis. The factors associated with thrombosis include the accumulation of uremic toxins and chronic low-grade inflammation, endothelial activation and impaired vascular integrity, a hypercoagulable state, and anemia (6).

CKD also plays a crucial role in the prognosis of patients with ACS. A study have pointed out that indicators reflecting renal function, such as creatinine, are closely related to the in-hospital mortality of patients with cardiogenic shock (7). Since ACS patients have a relatively high risk of developing cardiogenic shock, this means that the state of kidney disease is likely to affect the prognosis of cardiogenic shock in ACS patients. Meanwhile, the research by Mert İlker Hayıroğlu et al. (8) shows that impaired renal function increases the in-hospital death risk of patients with ST-segment elevation myocardial infarction (STEMI) complicated by cardiogenic shock. Since ACS includes some patients with STEMI, it further indicates that kidney disease cannot be ignored in the overall prognosis assessment of ACS. In clinical practice, ACS patients combined with CKD face a higher risk of adverse cardiovascular events, which highlights the necessity of formulating precise treatment strategies for such patients.

Although a significant portion and increasing number of the population diagnosed with ACS clinically has CKD, patients are often excluded from randomized trials. Guidelines still prioritize ticagrelor or prasugrel over clopidogrel in general population, there is currently no clear recommendation regarding which P2Y12 receptor antagonist should be chosen for patients with CKD. Given the high risk of adverse cardiovascular events and bleeding in the population with ACS combined with CKD, there is a need to develop appropriate antiplatelet treatment strategies.

Therefore, we conducted a systematic review and meta-analysis aimed at evaluating the comparative advantages of clopidogrel vs. ticagrelor in the choice of DAPT regimens for patients with ACS combined with CKD, while also exploring the appropriate duration of DAPT therapy in the presence of CKD.

## 2 Methods

The protocol of this studies was registered with PROSPERO (No. CRD42024593764). This review was conducted and reported according to the Preferred Reporting Items for a Systematic Review and Meta-analysis of Diagnostic Test Accuracy Studies (PRISMA-DTA) (9) and the PRISMA 2020 update (10).

### 2.1 Data sources and searches

The literature searches were conducted in the following four databases: PubMed, Embase, Cochrane Library, and Web of Science, covering publications from the inception of these databases up to September 1, 2024. For the search, we used both subject headings and free text terms, including “ST-elevation myocardial infarction,” “non-ST-elevation myocardial infarction,” “acute coronary syndrome,” “chronic kidney disease,” “antiplatelet therapy,” “aspirin,” “ticagrelor,” and “clopidogrel.” Additional searches were performed before the final analysis, with relevant studies identified for inclusion. Supplementary Table S1 showed detailed search strategies.

### 2.2 Eligibility criteria and study selection

Studies were considered eligible for inclusion if they met the following criteria: (1) Article type must be peer-reviewed journal articles to ensure the academic quality and reliability of the research; (2) The language is limited to English. This facilitates the unified acquisition and analysis scope of research data and avoids information bias caused by language barriers; (3) The research types are limited to cohort studies or randomized controlled trials; (4) The included patients must be clearly diagnosed with ACS combined with CKD. For the diagnosis of ACS, it should meet internationally recognized clinical diagnostic criteria, such as typical chest pain symptoms, dynamic electrocardiogram changes, and elevation of myocardial injury markers. The diagnosis of CKD is based on the eGFR. An eGFR <60 ml/min/1.73 m^2^ lasting for more than 3 months, or other evidence of kidney damage (such as proteinuria, hematuria, etc.) is present; (5) The intervention or control were confined to comparisons between different DAPT strategies; (6) At least one of the following outcomes was reported: all-cause mortality, major adverse cardiovascular events (MACE), major bleeding, acute myocardial infarction, cardiovascular death, stroke, and other adverse events with original data or risk ratios (RR), where MACE was defined as a total of several cardiovascular adverse events including acute myocardial infarction, cardiovascular death, and stroke.

The process of study selection was as follows: (1) Duplicate publications are excluded to avoid result bias caused by multiple inclusions of the same research data; (2) Non-cohort studies and non-randomized controlled trials are excluded because their research designs may not effectively control confounding factors, reducing the reliability of research conclusions; (3) Studies irrelevant to ACS combined with CKD are excluded to ensure that the included studies are closely related to the theme of this systematic review and meta-analysis; (4) Studies with inconsistent exposure factors (i.e., different DAPT strategies), study populations (such as large differences in patients' disease characteristics and baseline status), and outcome indicators are excluded to ensure the homogeneity of the included studies and improve the accuracy of the analysis results. Two independent reviewers (SL and DW) scanned the titles and abstracts according to the inclusion criteria. Any discrepancies regarding the search and selection were discussed in consultation with a third reviewer (GP) to reach a resolution. If a study appeared to potentially meet the inclusion criteria, the full text was retrieved for further evaluation.

### 2.3 Data extraction

After identifying the included studies, two reviewers (SL and DW) independently conducted data extraction. The extracted data included the following categories: (1) general information, including study name, publication year, study country, population, sample size, duration of follow-up, reported outcomes, and study type; (2) baseline information, including gender, age, proportion of hypertension, proportion of hyperlipidemia, proportion of diabetes, proportion of previous stroke, proportion of previous myocardial infarction, and proportion of previous percutaneous coronary intervention (PCI); (3) adjusted risk ratios (RR) and 95% confidence intervals (CI) for outcomes. After extraction, the data underwent thorough verification. Any discrepancies were verified and resolved by a third reviewer (GP). The study records were managed using EndNote 20 software. In this review, the management of missing values followed the approach reported in the original studies.

### 2.4 Outcomes and definitions

The determination of clinical outcomes was based on the assessment methods reported in the included original articles, with the outcomes reported in the original studies listed in Table 1. The primary outcome measures evaluated in this study were all-cause mortality, MACE, and major bleeding. The secondary outcomes included cardiovascular disease, acute myocardial infarction, and stroke. DAPT refers to the combination of aspirin with a P2Y12 receptor inhibitor.

**Table 1:** General characteristics of the included studies.

Order	Study ID	Country	Number of patients	Number of exposure cases	Number of control	Follow-up period (year)	Reported outcomes^a^	Study design
1	Stefan 2010	NR	3,237	NR	NR	0.9	①②③	Retrospective
2	Chien-Ho 2019	China	190	74	116	1	①②③④⑤⑥	Retrospective
3	Ji 2021	Korea	1,067	449	618	1	①②③④⑤⑥⑦⑧	Retrospective
4	Ying 2021	China	2,992	530	2,462	0.9	①③⑤⑥	Retrospective
5	Yun 2022	China	276	108	168	1	①②③④⑤⑥	Retrospective
6	Yun-S 2022	China	2,185	270	1,915	1	①②③④⑤⑥	Retrospective
7	Juan 2017	Sweden	7,348	3,839	3,509	1	①③⑤⑥⑨⑩	Prospective
8	Gargiulo 2017	Sweden	604	292	312	2	①③④⑤⑥	Prospective
9	Seokwoo 2020	Korea	2,246	516	1,730	1	①③⑤⑥⑨	Retrospective

NR, not reported.

^a^ Reported outcomes: ① all-cause mortality ② MACE ③ major bleeding ④ cardiovascular death ⑤ acute myocardial infarction ⑥ stroke ⑦ cerebrovascular accident ⑧ net adverse clinical events ⑨ REVASC, revascularization ⑩ ST, stent thrombosis.

### 2.5 Statistical analysis

#### 2.5.1 Data synthesis

A meta-analysis of six studies was conducted using RevMan 5.4.1 software. The overall effect size of all independent events was calculated using the combined RR. Data presented in the form of case numbers or risk ratios were converted to RR and 95% confidence intervals (95% CI) using the software's built-in calculation program. The *I*^2^ statistic was used to assess heterogeneity between studies, with *I*^2^values ≥50% indicating the presence of heterogeneity, in which case a random effects model was used; otherwise, a fixed effects model was applied. For results where fewer than four studies were reported, we did not perform a combined analysis. The *z* statistic for each outcome of interest was calculated, and results with a one-tailed *P* < 0.05 were considered statistically significant. The results of the meta-analysis were presented in the form of forest plots.

#### 2.5.2 Risk of bias

The risk of bias in cohort studies was independently assessed by two reviewers (SL and DW) using the Newcastle-Ottawa Scale (NOS), with scores ranging from 0 to 9 (11). For the follow-up duration in the NOS, we stipulated that a minimum of 6 months of follow-up was required to consider the occurrence of both primary and secondary outcomes.

Funnel plots generated using RevMan 5.4.1 software were used to assess publication bias. Publication bias was assessed by constructing a funnel plot to display the influence of individual studies on the outcomes of interest. The asymmetry of the funnel plot was also assessed using the Egger test (one-tailed *P* < 0.1 indicates significant publication bias). The assessment of publication bias was completed using STATA 14 software.

## 3 Results

### 3.1 Study selection

**Figure 1:**
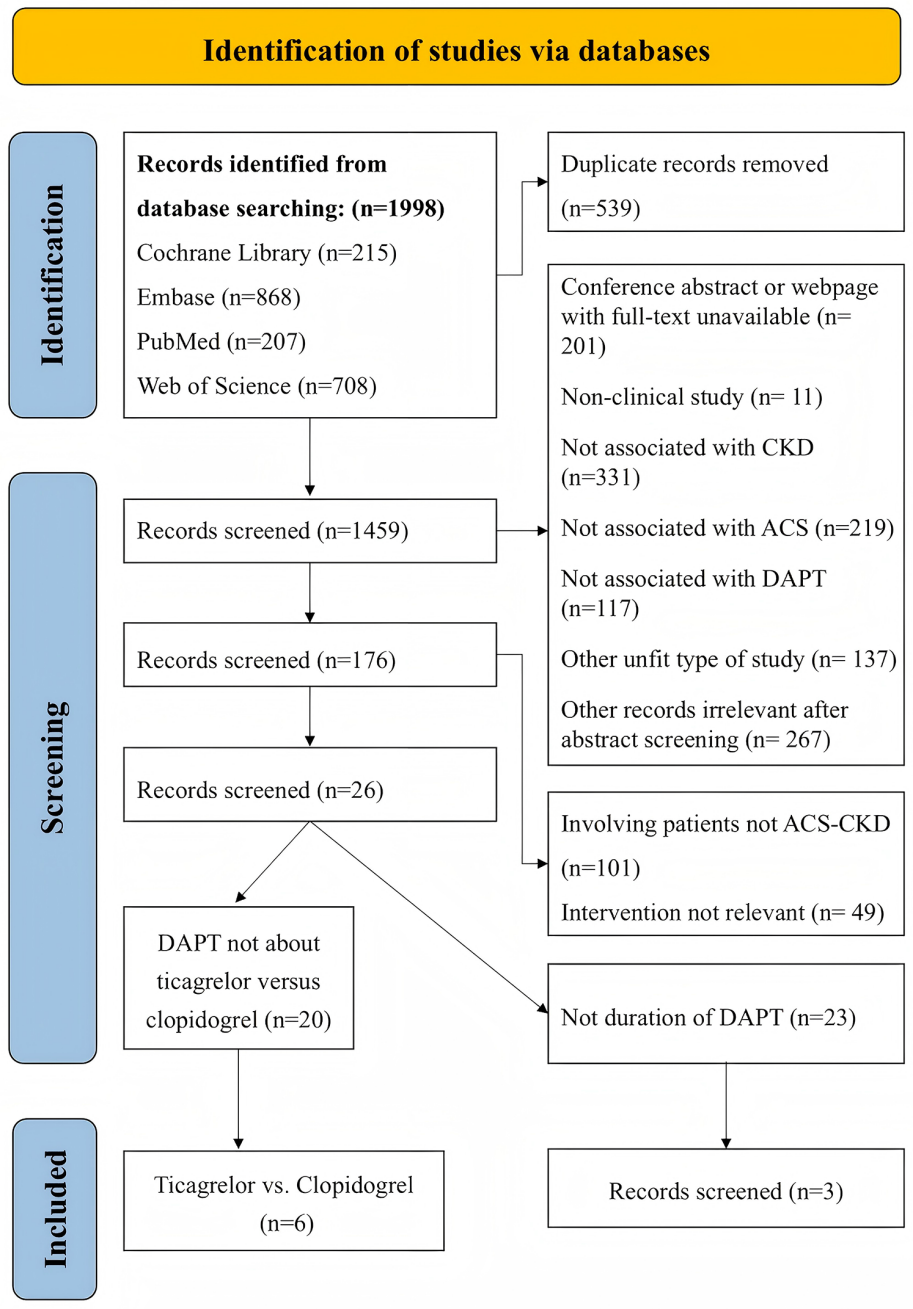
Flow chart of the article selection procedure for meta-analysis.

A total of 1,998 records were obtained from the initial search, and 539 duplicate records were removed. We screened the remaining 1,459 records by reading titles and abstracts, excluding 1,283 records that were deemed inappropriate article types or lacked relevance to ACS, CKD, and DAPT. After evaluating the full text of the remaining 176 articles, we excluded 101 records due to non-compliance with the disease criteria and 49 records due to differences in interventions. Ultimately, 26 studies were included, among which six articles (12–17) comparing ticagrelor to clopidogrel were included in the systematic review and meta-analysis, and three articles (18–20) regarding the duration of dual antiplatelet therapy were included in the systematic review. The process of study selection is visually depicted in Figure 1.

### 3.2 Study characteristics

**Figure 2:**
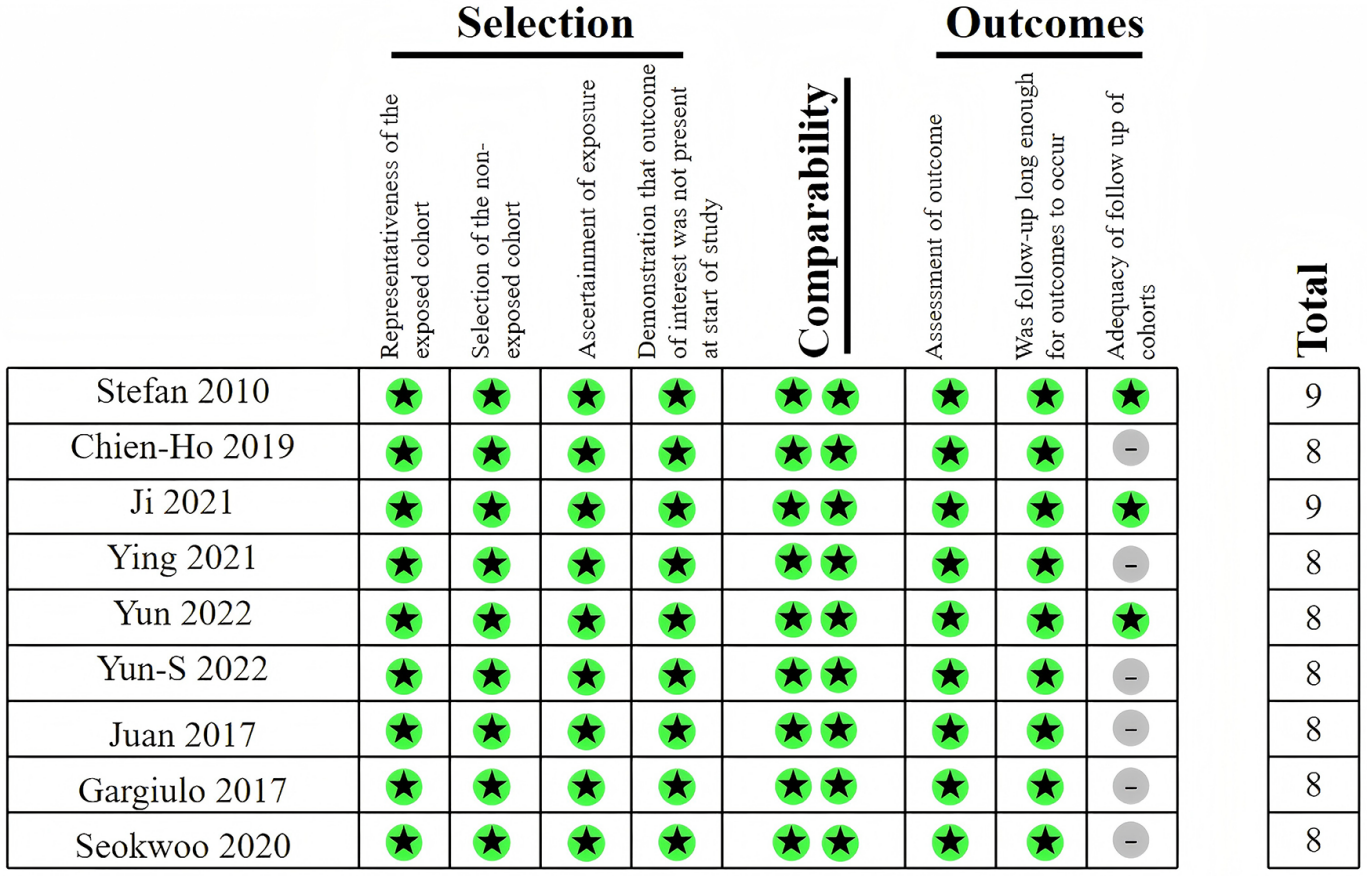
Newcastle-Ottawa scale for included studies in meta-analysis.

A total of 9,947 patients with ACS combined with CKD from six studies were included in the meta-analysis. All six studies were retrospective cohort studies. The duration of follow-up ranged from 9 months to 1 year. All six studies provided outcomes for all-cause mortality, major bleeding, and MACE. In terms of DAPT duration, one study (18) used 3 months as the time point, one study (19) compared 6 months vs. 24 months of drug use, and another study (20) involved durations of 12 months, 15 months, and 18 months. The general characteristics of the included studies are displayed in Table 1. Among the included studies, the NOS scores ranged from 8 to 9, as shown in Figure 2. The average age of the patients included in the nine studies was over 66 years, with a male preponderance and a high prevalence of comorbidities such as hypertension and hyperlipidemia. The baseline characteristics of the studies are displayed in Table 2. The detailed renal function of the studies are displayed in Table 3.

**Table 2:** Baseline characteristics of the included studies.

Order	Study ID	Age, year	Sex, male%	HT%	HL%	DM%	Previous stroke%	Previous AMI%	PCI%
1	Stefan 2010	74.00	60.20	77.80	47.80	33.00	6.60	28.30	15.80
2	Chien 2019	66.84	62.09	87.40	33.16	68.96	16.32	10.01	23.15
3	Ji 2021	69.00	75.15	78.90	58.85	61.00	13.85	11.15	22.10
4	Ying 2021	>75 (28.89%)	57.31	90.00	55.22	75.15	9.38	11.24	100.00
5	Yun 2022	67.46	69.63	76.10	NR	39.51	10.83	10.13	NR
6	Yun-S 2022	66.83	57.44	74.73	24.99	63.66	6.82	80.69	69.84
7	Juan 2017	77.17	54.53	68.37	32.20	24.20	5.69	17.10	11.31
8	Gargiulo 2017	77.63	63.12	79.83	51.81	27.67	0.00	59.27	20.54
9	Seokwoo 2020	77.63	63.12	79.83	51.81	27.67	0.00	59.27	20.54

HT, Hypertension; HL, Dyslipidemia; DM, Diabetes mellitus; AMI, Acute Myocardial Infarction; PCI, Percutaneous Coronary Intervention; NR, not reported.

**Table 3:** Detailed renal function of the included studies.

Order	Study ID	CKD stages	eGFR or CrCl	Proportion of dialysis patients
1	Stefan 2010	III–V	CrCl < 60 ml/min	NR
2	Chien-Ho 2019	V	eGFR < 15 ml/min/1.73 m^2^	100%
3	Ji 2021	III–V	eGFR < 60 ml/min/1.73 m^2^	23.3%
4	Ying 2021	V	eGFR < 15 ml/min/1.73 m^2^	100%
5	Yun 2022	IV–V	eGFR < 30 ml/min/1.73 m^2^	18.1%
6	Yun-S 2022	V	eGFR < 15 ml/min/1.73 m^2^	100%
7	Juan 2017	III–V	eGFR < 60 ml/min/1.73 m^2^	NR
8	Gargiulo 2017	III—V	eGFR < 60 ml/min/1.73 m^2^	NR
9	Seokwoo 2020	V	eGFR < 15 ml/min/1.73 m^2^	100%

NR, not reported.

### 3.3 Total results of meta-analysis

#### 3.3.1 Ticagrelor vs. clopidogrel

##### 3.3.1.1 Results of outcomes

**Figure 3:**
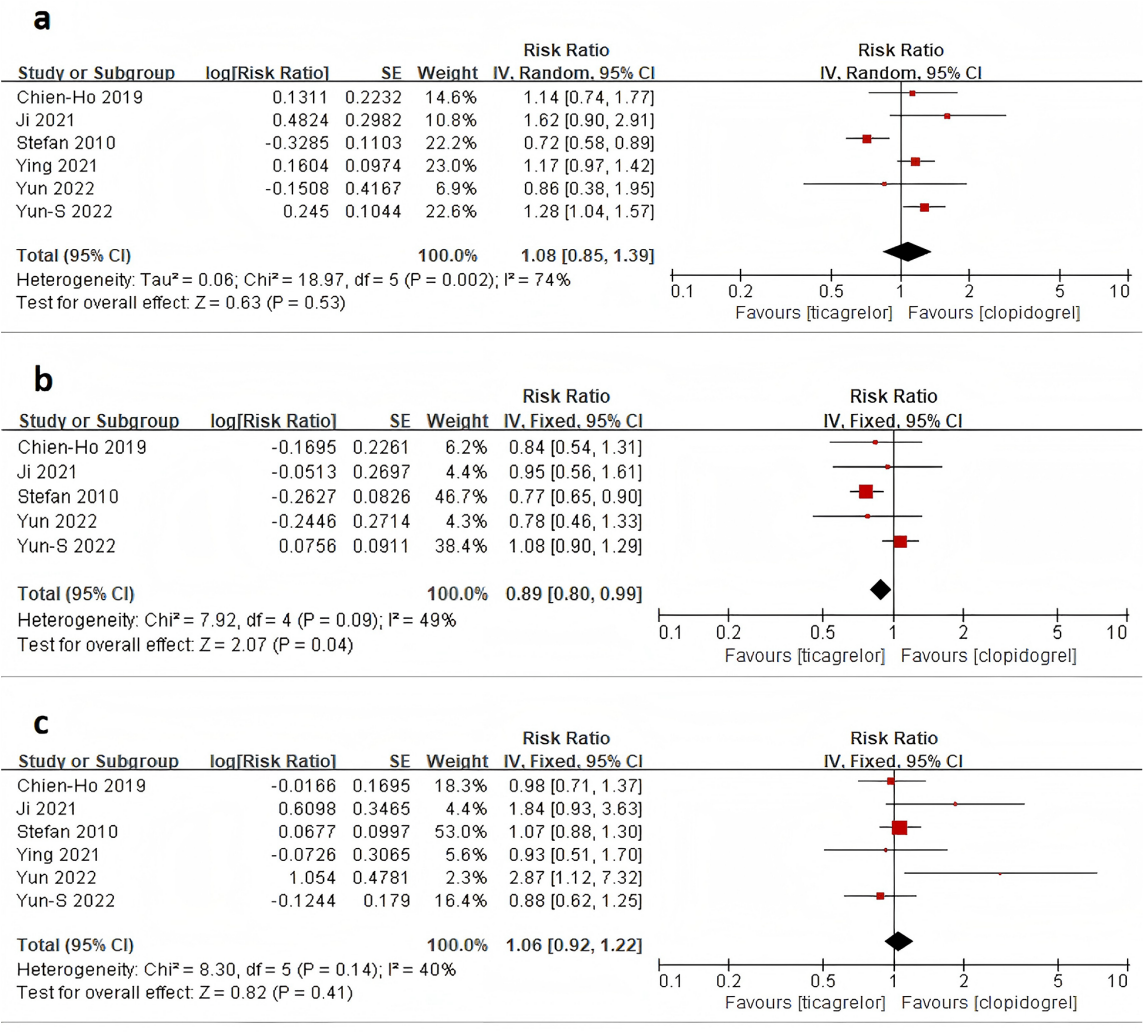
Forest plot of meta-analysis of studies involving primary endpoints between ticagrelor vs. Clopidogrel. **(a)** all-cause mortality; **(b)** MACE; **(c)** major bleeding.

All six studies reported the outcome of all-cause mortality. The heterogeneity analysis yielded an *I*^2^ value of 74% and a *P*-value of 0.53. Therefore, a meta-analysis using a random-effects model was conducted, with a combined RR of 1.08 (95% CI: 0.85–1.39), indicating that there was no statistically significant difference in all-cause mortality between treatment with ticagrelor and treatment with clopidogrel in patients with ACS combined with CKD (Figure 3a).

Five studies reported the outcome of MACE. The heterogeneity analysis yielded an *I*^2^ value of 49% and a *P*-value of 0.04. Therefore, a meta-analysis using a fixed-effects model was conducted, with a combined RR of 0.89 (95% CI: 0.80–0.99). The data indicate a lower rate of clinical MACE events in patients with ACS combined with CKD who received ticagrelor treatment compared to those who received clopidogrel (Figure 3b).

All six studies reported the outcome of major bleeding. The heterogeneity analysis yielded an *I*^2^ value of 40% and a *P*-value of 0.41. Therefore, a meta-analysis using a fixed-effects model was conducted, with a combined RR of 1.06 (95% CI: 0.92–1.22), indicating that there was no statistically significant difference in the risk of major bleeding between treatment with ticagrelor and treatment with clopidogrel in patients with ACS combined with CKD (Figure 3c).

Four studies reported the outcome of cardiovascular death, with a combined RR of 1.19 (95% CI: 0.94–1.51) and a *P*-value of 0.15 (Supplementary Figure S1A). Five studies reported the outcome of acute myocardial infarction, with a combined RR of 1.03 (95% CI: 0.79–1.35) and a *P*-value of 0.80 (Supplementary Figure S1B). For both of these outcomes, ticagrelor did not show a significant difference compared to clopidogrel. Four studies reported the outcome of stroke, with a combined RR of 0.66 (95% CI: 0.45–0.96) and a *P*-value of 0.03, indicating that patients with ACS and CKD receiving ticagrelor treatment had a lower rate of clinical stroke events compared to those receiving clopidogrel (Supplementary Figure S1C). Overview of all outcomes is shown in Table 4.

**Table 4:** Overview of all outcomes.

Outcomes	Studies	Effect estimate	*P*
All-cause mortality	6	1.08 [0.85, 1.39]	0.53
MACE	5	0.89 [0.80, 0.99]	0.04
Major bleeding	6	1.06 [0.92, 1.22]	0.41
Cardiovascular death	4	1.19 [0.94, 1.51]	0.15
Acute myocardial infarction	5	1.03 [0.79, 1.35]	0.80
Stroke	4	0.66 [0.45, 0.96]	0.03

##### 3.3.1.2 Publication bias

The funnel plot (Supplementary Figure S2) for the primary and secondary endpoints of six studies comparing ticagrelor vs. clopidogrel demonstrates symmetry, suggesting the absence of publication bias. Additionally, the results of the three primary endpoint indicators were subjected to the Egger's test using STATA 14 (Supplementary Figure S3), *t*_all−cause mortality_ = 0.19 (*P* = 0.860), *t*_MACE_ = 1.33 (*P* = 0.253), and *t*_major bleeding_ = −0.06 (*P* = 0.954) respectively, indicating no publication bias.

#### 3.3.2 The duration of DAPT

Three studies evaluated the impact of the duration of DAPT on clinical outcomes. Due to heterogeneity among the three studies (stemming from differences in intervention duration), a summary analysis was not conducted, and only the outcomes of individual studies are described.

In study Juan 2017 (18), the rate of all-cause mortality with DAPT for 3 months was higher (7.79% vs. 5.53%), as was the incidence of acute myocardial infarction (4.95% vs. 4.33%) and stroke (2.06% vs. 1.62%), compared to the group with DAPT lasting more than 3 months, while the incidence of major bleeding events was lower (1.48% vs. 1.97%). In study Gargiulo 2017 (19), the all-cause mortality rate (14.38% vs. 11.86%) and the incidence of acute myocardial infarction (8.22% vs. 6.41%) during 6 months of DAPT were higher compared to 24 months, while the risk of stroke (2.40% vs. 4.17%) and major bleeding (1.03% vs. 3.21%) was reduced. In study Seokwoo 2020 (20), comparing DAPT for more than one year, the all-cause mortality rate (10.27% vs. 5.95%) and the incidence of stroke (5.62% vs. 3.82%) were higher with one year of DAPT, while the rate of major bleeding events (4.46% vs. 6.36%) was lower, and there was no significant difference in the incidence of acute myocardial infarction (4.46% vs. 4.45%).

## 4 Discussion

### 4.1 Main findings

In this study, we compared the efficacy and safety of different DAPT strategies. We found that compared to clopidogrel-based DAPT, ticagrelor-based DAPT is associated with reduced MACE, which may be related to its inhibition of platelet activity and increased protection against stroke. The risk of major bleeding and all-cause mortality did not significantly increase. Meanwhile, as the duration of DAPT was prolonged, the all-cause mortality rate in patients significantly decreased, but the risk of major bleeding increased.

### 4.2 Interpretation

Since platelet aggregation is crucial in the progression and prognosis of ACS, DAPT is strongly recommended by guidelines as an essential component in the management of ACS. CKD can affect patients' platelet aggregation capacity and coagulation function, while the reduced renal excretory capacity can also influence the metabolism of antiplatelet medications. Therefore, patients with ACS and CKD have a significantly increased risk of both ischemia and bleeding, necessitating the development of personalized antiplatelet strategy in clinical practice to balance the risks of ischemia and bleeding.

The variability of platelet response to clopidogrel is influenced by genetic factors and drug interactions, which is particularly pronounced in patients with CKD. In CKD patients, the absorption, distribution, and metabolism of clopidogrel are impaired, significantly reducing its antiplatelet effect (21). Ticagrelor is metabolized and excreted by the kidneys to a very low extent, with the recovery rate of ticagrelor and its active metabolites in urine being less than 1% of the administered dose. Therefore, it is less affected by renal function. In patients with severe renal impairment, there are no statistically significant differences in the pharmacodynamics, pharmacokinetics, and safety data of ticagrelor compared to patients with normal renal function, suggesting that no dosage adjustment is necessary for ticagrelor in patients with severe renal impairment (22).

The TWILIGHT study evaluated the safety and efficacy of switching to ticagrelor monotherapy after a short-term DAPT in patients with complex lesions, diabetes, ACS, and different genders following PCI. However, it did not specifically investigate the safety and efficacy of ticagrelor compared to clopidogrel in patients with concurrent CKD (23). The PLATO sub-study on platelet inhibition indicated that ticagrelor provides more effective platelet inhibition than clopidogrel, regardless of the patient's resistance status to clopidogrel, both during maintenance therapy and in the first few hours of treatment. Additionally, ticagrelor acts more quickly than clopidogrel when used in ACS patients (24). The mechanisms by which clopidogrel and ticagrelor inhibit platelet aggregation are as follows: clopidogrel is an ADP receptor antagonist that inhibits the ADP receptor P2Y12 on the platelet surface, preventing ADP from binding to it, thereby reducing platelet aggregation and thrombosis (25). Ticagrelor inhibits the P2Y12 receptor through a non-competitive mechanism; under non-competitive binding conditions, increasing the concentration of ADP does not significantly alter ticagrelor's antiplatelet effect. This direct inhibitor can affect the externalized internal pool of P2Y12 receptors that are inaccessible during brief exposure to the thienopyridine and its metabolites (26). The mechanisms described above may help explain the lower incidence of ischemic events in the ticagrelor group compared to the clopidogrel group in the PLATO trial. This meta-analysis aims to clarify these uncertainties by summarizing clinical data regarding ticagrelor or clopidogrel in such specific patient populations. Our findings emphasize that ticagrelor offers greater benefits than clopidogrel in patients with ACS combined with CKD.

Regarding all-cause mortality, six studies indicated that the event rates for ticagrelor and clopidogrel were comparable, however, there was high heterogeneity (*I*^2^ = 74%). The main source of heterogeneity was the study by Stefan 2010, whose conclusions did not differ from the overall results. Excluding this study did not impact the overall results, and the source of heterogeneity may relate to the statistical methods employed. This study estimated the incidence rates of various outcomes based on the median follow-up time, however, it adjusted for confounding factors, reducing the risk of bias. We believe that including this study in the meta-analysis is appropriate. Since MACE is defined as a composite event of cardiovascular death, acute myocardial infarction, and stroke, the results from the analysis of secondary outcome measures indicate that the reduction in MACE with ticagrelor is primarily due to a decrease in the incidence of stroke. In terms of the risk of bleeding, there is no significant difference between ticagrelor and clopidogrel. Based on the above analysis, ticagrelor demonstrates greater benefits in patients with ACS combined with CKD compared to clopidogrel.

Regarding the duration of DAPT use, due to limitations in the study population and intervention measures, only three articles meeting the inclusion criteria were found, and therefore, a meta-analysis was not conducted. An analysis of the results from the included articles shows a consistent trend across all three studies, indicating that a strategy of extending the duration of DAPT may increase the clinical net benefits for patients. The overall mortality rate with DAPT exceeding one year is significantly reduced, and it does not increase the incidence of myocardial infarction and stroke events. Current consensus suggests that the benefits of antithrombotic therapy may diminish over time. The primary reason is that the risk of major bleeding increases with the duration of drug use. However, in patients with CKD, both bleeding risk and ischemic risk are significantly elevated. Therefore, overall mortality benefit seems to be the most important indicator for assessing benefits in this population. Our study results suggest that patients with ACS combined with CKD may benefit from a strategy of extending the duration of antithrombotic therapy, but the specific duration of DAPT still requires further confirmation.

When considering the potential subgroup differences, patients with different stages of CKD may respond differently to DAPT. For patients in the early stages of CKD, the kidney function is relatively better, and the impact of renal impairment on antiplatelet drug metabolism may be less significant. In contrast, patients with advanced CKD, especially those on dialysis, have severely impaired renal function. The accumulation of uremic toxins in these patients can further disrupt platelet function and coagulation pathways, potentially affecting the efficacy and safety of DAPT. For example, in patients with end-stage renal disease on dialysis, the risk of bleeding may be higher due to platelet dysfunction caused by uremia, and the choice between ticagrelor and clopidogrel may need to be more carefully considered. However, due to the limited number of studies and the combination of different CKD stages in our analysis, we were unable to draw definite conclusions about the differential treatment effects in each CKD stage.

Regarding the PCI status, patients who have undergone PCI often require more intensive antiplatelet therapy to prevent stent thrombosis. The choice between ticagrelor and clopidogrel may also be influenced by the type of stent used and the time since PCI. In our meta-analysis, we did not stratify the results based on PCI status, which is a limitation. Future studies should focus on exploring the optimal DAPT strategy in ACS patients with CKD who have undergone PCI, taking into account factors such as the timing of PCI, stent type, and renal function.

### 4.3 Clinical application suggestion

The results of this study are of clinical significance. In the treatment of patients with ACS combined with CKD, it has been established that ticagrelor-based DAPT has a distinct advantage over clopidogrel in reducing the incidence of MACE. This provides crucial evidence-based medical evidence for clinicians when formulating treatment plans. Currently, clinical treatment decisions for this high-risk patient group face numerous formidable challenges. However, the conclusion of this study can effectively guide clinicians to prioritize the use of ticagrelor in DAPT. This contributes to reducing the risk of cardiovascular events in patients and further improving their clinical outcomes.

At the same time, the study has found that shortening the DAPT treatment course does not improve the clinical prognosis of patients. This result has direct and clear guiding value for clinical practice and can effectively prevent potential adverse consequences that may be caused by clinicians and patients wrongly shortening the treatment course. This finding also points the way for further exploration of an appropriate DAPT treatment course, driving research in related fields to focus on how to achieve a reasonable balance between bleeding risk and ischemic risk while ensuring treatment effectiveness, and to formulate more precise and personalized treatment plans. Through these efforts, the overall treatment level of patients with ACS combined with CKD can be significantly enhanced, patient mortality can be reduced, and the quality of life of patients can be improved.

### 4.4 Comparison with previous studies

A recent double-blind (27) randomized controlled trial comparing the effects of ticagrelor and clopidogrel in 48 patients with CKD showed that ticagrelor is superior to clopidogrel in inhibiting platelet aggregation in patients with stages 4–5 CKD. This finding is consistent with our study conclusions. Moreover, in addition to better platelet inhibition, ticagrelor also elicited a more favorable anti-inflammatory response compared to clopidogrel in asymptomatic patients with stages 4–5 CKD. A previous meta-analysis (28) investigating potent P2Y12 inhibitors compared to clopidogrel in patients with ACS combined with CKD pointed out that potent P2Y12 inhibitors lead to a reduction in the incidence of MACE without an increase in the rate of major bleeding. These findings are consistent with our meta-analysis. Based on previous data, our study includes articles from 2010 to the present to further support the above findings. Other studies also confirmed this viewpoint (29).

In addition, an observational study (30) conducted in Canada on the use of DAPT in patients with ACS combined with CKD indicated that, compared to patients without CKD, those with CKD are less likely to receive potent P2Y12 inhibitors, have a shorter duration of DAPT treatment, and have a higher bleeding risk. A previous meta-analysis (31) comparing the duration of DAPT in patients with CKD found that DAPT duration exceeding 6 months was associated with a lower rate of the composite outcomes of all-cause mortality, myocardial infarction, stroke, stent thrombosis, or major bleeding compared to DAPT lasting 12 months. This contrasts with the findings of our study, and the difference may be attributable to the fact that the study population was not confined to ACS. Compared to patients with ACS, those with chronic coronary syndrome derive less benefit from DAPT. Analysis of the PEGASUS-TIMI 54 trial indicates that although the discontinuation rate is high, extending DAPT with aspirin and ticagrelor for 12 months post-acute coronary syndrome is associated with a reduction in the absolute risk of cardiovascular mortality, myocardial infarction, or stroke. Furthermore, in patients with CKD, this risk reduction is increased fourfold (32). This is consistent with the conclusions of our study.

### 4.5 Limitations

Our study has several limitations. First and foremost, all six studies included in the meta-analysis were retrospective cohort studies. Retrospective cohort studies inherently suffer from the issue of baseline imbalance, despite the efforts of multivariable adjustments, it is impossible to completely eliminate the bias resulting from baseline differences. For example, unmeasured confounding factors such as patients' lifestyle habits, genetic predispositions, and environmental exposures could influence the outcomes but are difficult to account for in retrospective designs.

The heavy reliance on retrospective cohort studies is mainly due to the lack of well-designed randomized controlled trials (RCTs) in this field. However, in the context of ACS combined with CKD, it is challenging to conduct RCTs. The complex comorbidities and high-risk nature of these patients make it difficult to meet the strict inclusion and exclusion criteria often required in RCTs. Also, ethical considerations may limit the randomization of patients to different antiplatelet regimens, especially when one treatment option may be perceived as more beneficial based on existing evidence.

Although our meta-analysis shows that ticagrelor-based DAPT has advantages over clopidogrel-based DAPT in reducing the incidence of MACE, the lack of RCTs weakens the strength of our evidence. A recent real-world study comparing ticagrelor and clopidogrel reported similar results to our study, but it focused on a different patient population (33). This real-world study may have different selection biases and confounding factors compared to our meta-analysis. While the consistency in results is encouraging, it also highlights the need for more RCTs to confirm these findings in a more rigorous and controlled setting.

Another limitation is that the analysis did not include the use of other cardiovascular medications during the study. Different medications, such as statins, beta-blockers, and anticoagulants, can interact with antiplatelet drugs and affect the occurrence of adverse cardiovascular events and bleeding. Additionally, the consideration of whether patients underwent PCI and the impact of PCI on adverse cardiovascular outcomes were not taken into account, which could also affect the results; however, this study is a study-level meta-analysis and cannot distinguish a subgroup analysis for patients who underwent PCI.

Finally, the analysis combined patients with different stages of CKD, including those on dialysis for end-stage renal disease, as well as patients with CKD stages III and IV. The pathophysiology and treatment responses may vary significantly among different CKD stages. Combining these patients may mask the true treatment effects in specific CKD subgroups.

## 5 Conclusions

The evidence from this meta-analysis indicates that in patients with ACS combined with CKD, ticagrelor-based DAPT has advantages over clopidogrel-based DAPT, which is associated with a lower incidence of MACE. In patients with ACS combined with CKD, shortening the duration of antithrombotic therapy does not improve clinical outcomes. We highlight the need for further research on the duration of DAPT in patients with ACS combined with CKD.
