# Supplementary material for: Antiplatelet strategy for patients with acute coronary syndrome and chronic kidney disease: a systematic review and meta-analysis
Source: Front Cardiovasc Med. 2025 Feb 20;12:1527667. doi: 10.3389/fcvm.2025.1527667 (PMC11882542; doi:10.3389/fcvm.2025.1527667)
Supplement: Supplementary file 1 [file Datasheet.pdf]

frontiers

Supplementary Material

Antiplatelet strategy for patients with acute coronary syndrome and chronic kidney disease: a systematic review and meta-analysis

Siqi Li[1†], Dayang Wang [2,3†], Xiaowan Han[2], Diying Zhang[1], Hongxiao Deng[1], Guozhong Pan[2*]

*** Correspondence to:** Guozhong Pan: panguozhong108@sina.com

1 Supplementary Figures and Tables

1.1 Supplementary Figures

1.1.1 Supplementary FigureS1

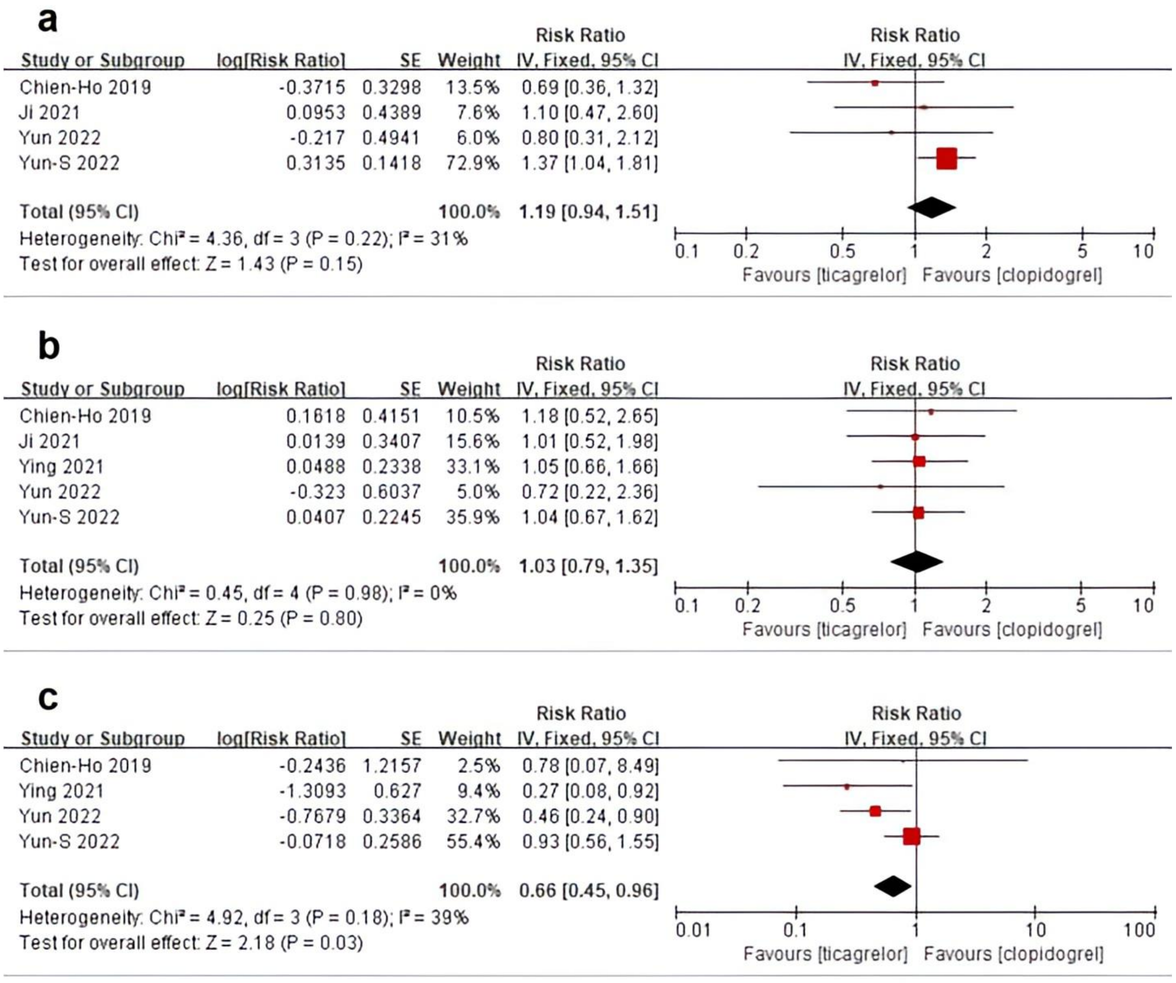

Figure S1. Forest plot of meta-analysis of studies involving secondary endpoints between Ticagrelor vs. Clopidogrel. (a) cardiovascular death; (b) acute myocardial infarction;(c) stroke

1.1.2 Supplementary FigureS2

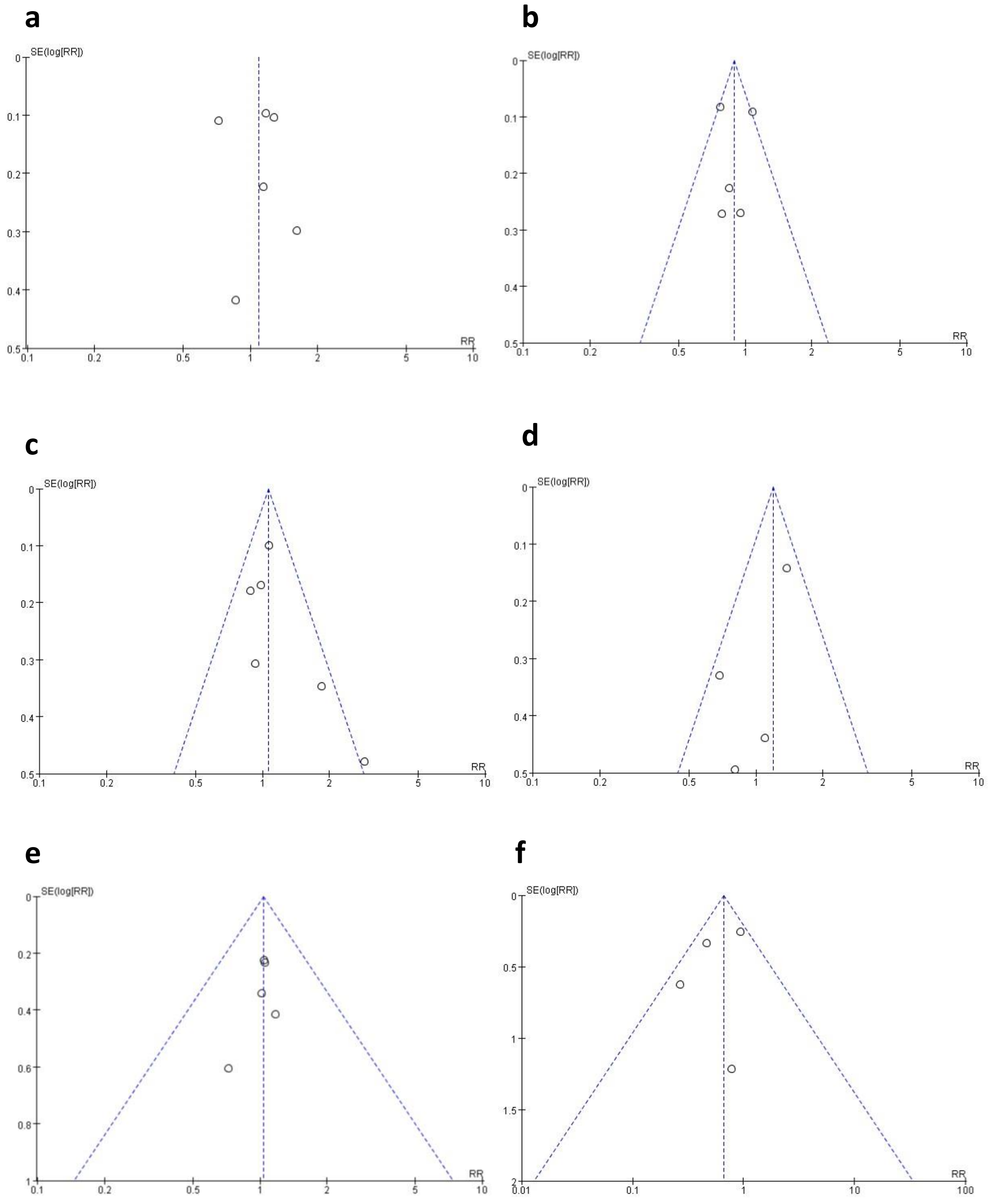

Figure S2. Funnel plots for outcomes. (a) all-cause mortality. (d) MACE. (c) major bleeding. (d) cardiovascular death. (e) myocardial infarction. (f) stroke.

1.1.3 Supplementary FigureS3

a

```
. metabias log selog, graph egger

Note: data input format theta se_theta assumed.

Egger's test for small-study effects:
Regress standard normal deviate of intervention
effect estimate against its standard error

Number of studies =   6                               Root MSE      =    2.168
```

Std_Eff	Coef.	Std. Err.	t	P>|t|	[95% Conf.	Interval]
slope	.0151284	.2775312	0.05	0.959	-.7554217	.7856784
bias	.3777162	2.01547	0.19	0.860	-5.218125	5.973558

```
Test of H0: no small-study effects          P = 0.860
```

b

```
.  metabias log selog, graph egger

Note: data input format theta se_theta assumed.

Egger's test for small-study effects:
Regress standard normal deviate of intervention
effect estimate against its standard error

Number of studies =   6                               Root MSE      =    1.199
```

Std_Eff	Coef.	Std. Err.	t	P>|t|	[95% Conf.	Interval]
slope	-.1569691	.1840838	-0.85	0.442	-.6680678	.3541296
bias	1.380763	1.03581	1.33	0.253	-1.495107	4.256634

```
Test of H0: no small-study effects          P = 0.253
```

c

```
.  metabias log selog, graph egger

Note: data input format theta se_theta assumed.

Egger's test for small-study effects:
Regress standard normal deviate of intervention
effect estimate against its standard error

Number of studies =   5                               Root MSE      =    1.623
```

Std_Eff	Coef.	Std. Err.	t	P>|t|	[95% Conf.	Interval]
slope	-.1062933	.1935164	-0.55	0.621	-.7221488	.5095621
bias	-.096432	1.53342	-0.06	0.954	-4.976458	4.783594

```
Test of H0: no small-study effects          P = 0.954
```

Figure S3.Publication bias assessment of meta-analysis. (a) all-cause mortality. (d) MACE. (c) major bleeding

1.2 Supplementary TableS1

Search Strategy

PubMed (n=207)	((ST Elevation Myocardial Infarction[Title/Abstract]) OR (STEMI[Title/Abstract]) OR (ST Segment Elevation Myocardial Infarction[Title/Abstract]) OR (ST Elevated Myocardial Infarction[Title/Abstract]) OR (Non-ST Elevated Myocardial Infarction[Title/Abstract]) OR (Non ST Elevated Myocardial Infarction[Title/Abstract]) OR (NSTEMI[Title/Abstract]) OR (Non-ST-Elevation Myocardial Infarction[Title/Abstract]) OR (Infarction, Non-ST-Elevation Myocardial[Title/Abstract]) OR (Infarctions, Non-ST-Elevation Myocardial[Title/Abstract]) OR (Myocardial Infarction, Non-ST-Elevation[Title/Abstract]) OR (Myocardial Infarctions, Non-ST-Elevation[Title/Abstract]) OR(Non ST Elevation Myocardial Infarction[Title/Abstract]) OR (coronary artery disease[Title/Abstract]) OR (acute myocardial infarction[Title/Abstract]) OR (AMI[Title/Abstract]) OR (acute coronary syndrome [Title/Abstract]) OR (ACS[Title/Abstract]))<br>AND<br>((Renal Insufficiency, Chronic[Title/Abstract]) OR (Chronic Renal Insufficiencies[Title/Abstract]) OR (Renal Insufficiencies, Chronic[Title/Abstract]) OR (Chronic Kidney Insufficiency[Title/Abstract]) OR (Chronic Kidney Insufficiencies[Title/Abstract]) OR (Kidney Insufficiencies, Chronic[Title/Abstract]) OR (Chronic Renal Insufficiency[Title/Abstract]) OR (Kidney Insufficiency, Chronic[Title/Abstract]) OR (Chronic Kidney Diseases[Title/Abstract]) OR (Chronic Kidney Disease [Title/Abstract]) OR (Disease, Chronic Kidney[Title/Abstract]) OR (Diseases, Chronic Kidney[Title/Abstract]) OR (Kidney Disease, Chronic[Title/Abstract]) OR (Kidney Diseases, Chronic[Title/Abstract]) OR (Chronic Renal Diseases[Title/Abstract]) OR (Chronic Renal Disease[Title/Abstract]) OR (Disease, Chronic Renal[Title/Abstract]) OR (Diseases, Chronic Renal[Title/Abstract]) OR (Renal Disease, Chronic[Title/Abstract]) OR (Renal Diseases, Chronic[Title/Abstract]) OR<br>(Renal Diseases, Chronic[Title/Abstract]) OR (CKD[Title/Abstract]))<br>AND<br>((Antiplatelet [Title/Abstract]) OR (Antiplatelet Therapy[Title/Abstract]) OR (Anti-Platelet Therapies, Dual[Title/Abstract]) OR (Anti-Platelet Therapy, Dual[Title/Abstract]) OR(Dual Anti-Platelet Therapies[Title/Abstract]) OR (Dual Anti Platelet Therapy[Title/Abstract]) OR (DAPT[Title/Abstract]) OR (p2y12[Title/Abstract]) OR (Aspirin [Title/Abstract]) OR (clopidogrel [Title/Abstract]) OR (ticagrelor [Title/Abstract]) OR (Prasugrel

	[Title/Abstract])) NOT ((Review[Filter]) OR (Meta-Analysis [Filter]) OR(Case Reports [Filter]) OR (Books and Documents [Filter]) OR (Editorial[Filter]))
Embase (n=868)	#1 'st segment elevation myocardial infarction'/exp<br>#2 ('st segment elevation myocardial infarction':ti,ab,kw OR stemi:ti,ab,kw OR 'st elevation myocardial infarction':ti,ab,kw OR 'st elevated myocardial infarction':ti,ab,kw OR 'non-st elevated myocardial infarction':ti,ab,kw OR nstemi:ti,ab,kw OR 'non-st-elevation myocardial infarction':ti,ab,kw OR 'infarction, non-st-elevation myocardial':ti,ab,kw) AND 'infarctions, non-st-elevation myocardial':ti,ab,kw OR 'myocardial infarction, non-st-elevation':ti,ab,kw OR 'myocardial infarctions, non-st-elevation':ti,ab,kw OR 'non st elevation myocardial infarction':ti,ab,kw OR 'coronary artery disease':ti,ab,kw OR 'acute myocardial infarction':ti,ab,kw OR ami:ti,ab,kw OR 'acute coronary syndrome':ti,ab,kw OR acs:ti,ab,kw OR 'non st elevated myocardial infarction':ti,ab,kw<br>#3 #1 OR #2<br>#4 'chronic kidney failure'/exp<br>#5 ('chronic kidney failure':ti,ab,kw OR 'renal insufficiency, chronic':ti,ab,kw OR 'chronic renal insufficiencies':ti,ab,kw OR 'renal insufficiencies, chronic':ti,ab,kw OR 'chronic kidney insufficiency':ti,ab,kw OR 'chronic kidney insufficiencies':ti,ab,kw OR 'kidney insufficiencies, chronic':ti,ab,kw OR 'chronic renal insufficiency':ti,ab,kw) AND 'kidney insufficiency, chronic':ti,ab,kw OR 'chronic kidney diseases':ti,ab,kw OR 'chronic kidney disease':ti,ab,kw OR 'disease, chronic kidney':ti,ab,kw OR 'diseases, chronic kidney':ti,ab,kw OR 'kidney disease, chronic':ti,ab,kw OR 'kidney diseases, chronic':ti,ab,kw OR 'chronic renal diseases':ti,ab,kw OR 'chronic renal disease':ti,ab,kw OR 'disease, chronic renal':ti,ab,kw OR 'diseases, chronic renal':ti,ab,kw OR 'renal disease, chronic':ti,ab,kw OR 'renal diseases, chronic':ti,ab,kw OR ckd:ti,ab,kw<br>#6 #4 OR #5<br>#7 'dual antiplatelet therapy'/exp<br>#8 ('dual antiplatelet therapy':ti,ab,kw OR antiplatelet:ti,ab,kw OR 'antiplatelet therapy':ti,ab,kw OR 'anti-platelet therapies, dual':ti,ab,kw OR 'anti-platelet therapy, dual':ti,ab,kw OR 'dual anti-platelet therapies':ti,ab,kw OR 'dual anti platelet therapy':ti,ab,kw OR dapt:ti,ab,kw) AND p2y12:ti,ab,kw OR aspirin:ti,ab,kw OR clopidogrel:ti,ab,kw OR ticagrelor:ti,ab,kw OR prasugrel:ti,ab,kw<br>#9 #7 OR #8<br>#10 #3 AND #6 AND #9 NOT ('case report'/de OR 'nonhuman'/de OR 'meta analysis'/de OR 'systematic review'/de)
Cochrane	#1 MeSH descriptor: [ST Elevation Myocardial Infarction] explode all trees

Library (n=215)	#2 (STEMI):ti,ab,kw OR (ST Segment Elevation Myocardial Infarction):ti,ab,kw OR (ST Elevated Myocardial Infarction):ti,ab,kw OR (Non-ST Elevated Myocardial Infarction):ti,ab,kw OR (Non ST Elevated Myocardial Infarction):ti,ab,kw OR<br>(NSTEMI):ti,ab,kw OR (Non-ST-Elevation Myocardial Infarction):ti,ab,kw OR (Infarction, Non-ST-Elevation Myocardial):ti,ab,kw OR (Infarctions, Non-ST-Elevation Myocardial):ti,ab,kw OR (Myocardial Infarction, Non-ST-Elevation):ti,ab,kw OR (Myocardial Infarctions, Non-ST-Elevation):ti,ab,kw OR (Non ST Elevation Myocardial Infarction):ti,ab,kw OR (coronary artery disease):ti,ab,kw OR (acute myocardial infarction):ti,ab,kw OR (AMI):ti,ab,kw OR (acute coronary syndrome):ti,ab,kw OR (ACS):ti,ab,kw<br>#3 #1 OR #2<br>#4 MeSH descriptor: [Renal Insufficiency, Chronic] explode all trees<br>#5 (Chronic Renal Insufficiencies):ti,ab,kw OR (Renal Insufficiencies, Chronic):ti,ab,kw OR (Chronic Kidney Insufficiency):ti,ab,kw OR (Chronic Kidney Insufficiencies):ti,ab,kw OR (Kidney Insufficiencies, Chronic):ti,ab,kw OR (Chronic Renal Insufficiency):ti,ab,kw OR (Kidney Insufficiency, Chronic):ti,ab,kw OR (Chronic Kidney Diseases):ti,ab,kw OR (Chronic Kidney Disease):ti,ab,kw OR (Disease, Chronic Kidney):ti,ab,kw OR (Diseases, Chronic Kidney):ti,ab,kw OR (Kidney Disease, Chronic):ti,ab,kw OR (Kidney Diseases, Chronic):ti,ab,kw OR (Chronic Renal Diseases):ti,ab,kw OR (Chronic Renal Disease):ti,ab,kw OR (Disease, Chronic Renal):ti,ab,kw OR (Diseases, Chronic Renal):ti,ab,kw OR (Renal Disease, Chronic):ti,ab,kw OR (Renal Diseases, Chronic):ti,ab,kw OR (CKD):ti,ab,kw<br>#6 #4 OR #5<br>#7 MeSH descriptor: [Dual Anti-Platelet Therapy] explode all trees<br>#8 (Antiplatelet):ti,ab,kw OR (Antiplatelet Therapy):ti,ab,kw OR(Anti-Platelet Therapies, Dual):ti,ab,kw OR (Anti-Platelet Therapy, Dual):ti,ab,kw OR (Dual Anti-Platelet Therapies):ti,ab,kw OR (Dual Anti Platelet Therapy):ti,ab,kw OR (DAPT):ti,ab,kw OR (p2y12):ti,ab,kw OR (Aspirin):ti,ab,kw OR (clopidogrel):ti,ab,kw OR (ticagrelor):ti,ab,kw OR (Prasugrel):ti,ab,kw<br>#9 #7 OR #8<br>#10 #3 AND #6 AND #9
Web of Science (n=708)	TS=(ST Elevation Myocardial Infarction OR STEMI OR ST Segment Elevation Myocardial Infarction OR ST Elevated Myocardial Infarction OR Non-ST Elevated Myocardial Infarction OR Non ST Elevated Myocardial Infarction OR<br>NSTEMI OR Non-ST-Elevation Myocardial Infarction OR Infarction, Non-ST-Elevation Myocardial OR Infarctions, Non-ST-Elevation Myocardial OR Myocardial Infarction, Non-ST-Elevation OR Myocardial Infarctions, Non-ST-Elevation ORNon ST Elevation Myocardial Infarction OR coronary artery disease OR acute myocardial infarction OR AMI ORacute coronary syndrome

	ORACS)<br>AND<br>TS= (Renal Insufficiency, Chronic OR Chronic Renal Insufficiencies OR Renal Insufficiencies, Chronic OR Chronic Kidney Insufficiency OR Chronic Kidney Insufficiencies OR Kidney Insufficiencies, Chronic OR Chronic Renal Insufficiency OR Kidney Insufficiency, Chronic OR Chronic Kidney Diseases OR Chronic Kidney Disease OR Disease, Chronic Kidney OR Diseases, Chronic Kidney OR Kidney Disease, Chronic OR Kidney Diseases, Chronic OR Chronic Renal Diseases OR Chronic Renal Disease OR Disease, Chronic Renal OR Diseases, Chronic Renal OR Renal Disease, Chronic OR Renal Diseases, Chronic OR CKD)<br>AND<br>TS= (Antiplatelet OR Antiplatelet Therapy ORAnti-Platelet Therapies, Dual OR Anti-Platelet Therapy, Dual OR Dual Anti-Platelet Therapies OR Dual Anti Platelet Therapy OR DAPT OR p2y12 OR Aspirin OR clopidogrel OR ticagrelor OR Prasugrel)<br>NOT<br>DT=(case report OR nonhuman OR meta analysis OR systematic review)
